# Pokorny's complaint: the insoluble problem of the overwhelming number of false positives generated by suicide risk assessment

**DOI:** 10.1192/pb.bp.115.053017

**Published:** 2017-02

**Authors:** Olav Nielssen, Duncan Wallace, Matthew Large

**Affiliations:** 1 St Vincent's Hospital, Sydney, Australia; 2 School of Psychiatry, University of New South Wales, Australia

## Abstract

Alex Pokorny's 1983 prospective study of suicide found that 96.3% of high-risk predictions were false positives, and that more than half of the suicides occurred in the low-risk group and were hence false negatives. All subsequent prospective studies, including the recent US Army Study To Assess Risk and Resilience in Servicemembers (STARRS), have reported similar results. We argue that since risk assessment cannot be a practical basis for interventions aimed at reducing suicide, the alternative is for mental health services to carefully consider what amounts to an adequate standard of care, and to adopt the universal precaution of attempting to provide that to all of our patients.

## Pokorny's pioneering study

It is over 30 years since the publication of Alex Pokorny's landmark prospective study of suicide among consecutive first admissions to the Houston Veterans Administration Medical Center.^1,2^ Pokorny examined a cohort of 4800 men using a comprehensive array of relevant and reliable rating scales and assessed 803 (17%) to be at high risk of suicide because of the presence of a combination of risk factors. In the next 5 years, 30 (3.7%) high-risk patients and 37 (0.9%) of the 3997 lower-risk patients died by suicide, an odds of suicide that was 4 times greater in the high-risk group.

The 37 suicides among lower-risk patients were in effect false negatives. Pokorny considered false negatives to be inevitable because patients sometimes conceal their suicidal plans and their circumstances change over time. What concerned him more was the overwhelming proportion of false positives, because 96.3% of the patients categorised as high-risk did not commit suicide. He concluded:

‘We might tolerate 50% false negatives; if we could apply a screening test that would correctly identify only half of the future suicides without false positives that would be very helpful. However, with currently known tests to identify the actual suicides, we will also have to make a great many false-positive identifications, labeling up to a quarter of the total group as future suicides when only 1% to 5% actually are. From a cost-benefit standpoint, the application of such a test is simply not feasible.’^1^

## Other prospective studies of suicide

There have been relatively few prospective studies of the factors associated with subsequent suicide, probably because of the methodological and logistical difficulties involved. Not long after Pokorny, Goldstein *et al*^3^ followed up a cohort of 1906 patients diagnosed with affective disorders and found that none of the 46 suicides occurred among the group, albeit smaller than in Pokorny's study, defined as being at high risk. More recently, two prospective studies examined the proportion of suicide deaths among those considered to be at high risk.^4–6^ Madsen *et al*^4,5^ reported a suicide rate of 0.14% among those defined as high risk in a national study of the suicide of psychiatric in-patients in Denmark, and Steeg reported the suicide of 0.5% of patients identified as being at high risk among a large cohort of people who presented to emergency departments in three English counties after self-harm.^6^

Faced with growing concern about suicides among veterans and current serving members of the U.S. Armed Forces, the U.S. Department of Veterans Affairs and the U.S. Department of Defense included the use of risk stratification in clinical practice guidelines.^7^ As part of the Study to Assess Risk and Resilience in Servicemembers (STARRS), Kessler and associates then examined factors associated with suicide among 53 769 American soldiers in the 12 months after discharge from military psychiatric hospitals,^8^ with the benefit of the very detailed longitudinal US Army personnel database and highly sophisticated statistical techniques derived from artificial intelligence research. They identified a high-risk group comprising 2689 admissions, or 5% of the sample, from which there were 36 suicides, corresponding to a 22 times increased risk of suicide in that group. However, in other respects the results were eerily familiar, as nearly half of the suicides occurred among the 51 080 low-risk patients, and only 1.3% of the high-risk group died by suicide, leaving an overwhelming number of false positive cases.

The problem remains of the disturbingly high suicide rates among psychiatric patients as a whole. For example, in the Madsen study, the rate of suicide of psychiatric in-patients was 72 times that of the general community, at 860 per 100 000 patient years, and in the Steeg study the suicide rate among all patients presenting to hospital after self-harm was 622 per 100 000 patient years. Hence, although we know that all of the patients under our care are at greatly increased risk of suicide compared with the wider community,^5^ our ability to distinguish between groups of patients with respect to the probability of subsequent suicide risk is, at best, quite modest.

## Implications for mental health services

This observation has important implications for mental health services, given the unrealistic expectations for risk assessment to prevent serious adverse events, and the way risk assessment has changed professional practice.^9^ If risk stratification is to be a part of how mental health services approach suicide prevention, we need to carefully consider the interventions offered to patients believed to be at high risk but denied to those assessed to be at lower risk. Because the vast majority of high-risk patients will not die by suicide, any intervention has to be shown to be somewhat effective, but also benign enough so as not to infringe on the rights of the many with false positive assessments. However, if we do have an effective and benign intervention, it is then hard to justify denying this to low-risk patients, who, after all, are still many times more likely to die by suicide than the general community.

If there is no effective and yet benign intervention to justify offering different treatments to groups of patients on the basis of risk stratification, how can mental health services respond to the knowledge that our patients are at greatly increased risk of suicide compared with the wider community?

The alternative is the application of universal precautions to all patients, including the design of in-patient facilities that minimise the opportunity to attempt suicide,^10^ the provision of community treatment for all patients in the weeks after discharge from hospital,^11^ and safety planning at the point of contact in emergency departments.^12^ Most importantly, every patient should have access to timely, individualised, high-quality treatment for psychiatric disorder. Modifiable factors associated with suicide should be addressed in all patients, not only those considered to be at greatest risk. Moreover, no intervention can be justified on the basis of the assessed risk alone. We go so far as to suggest that the assessed risk of suicide on its own is not a sufficient reason for restrictive interventions such as involuntary detention in hospital or other coercive treatment,^13,14^ which would then need to be justified on additional grounds, including the patient's inability to recognise the potential benefit of treatment and their particular circumstances and treatment needs. In any case, preventive detention in hospital of all patients assessed to be at high risk of suicide would be impossible, because of the huge numbers of patients identified and the long duration of secure detention that would be required to protect such patients.

Addressing the modifiable risk factors in populations of patients involves ensuring the adequate identification, assessment and treatment of patients with a range of mental disorders, addressing substance use and, wherever possible, limiting the availability of potentially lethal means to die by suicide. Treatment should be with the patient's consent, or provided on the basis that the patient lacks the capacity to consent, rather than being based on perceived risk, which we now know we are not able to reliably assess.

A further implication for mental health services of the demonstrated limitation of risk assessment is in responding to lawsuits for failing to predict the suicides of individual patients. While the suicide of any patient might be foreseeable in the legal sense of being not fanciful or far-fetched, mental health services cannot be reasonably expected to be able to identify which patients will die by suicide. Expert evidence in one recent case on the estimated probability of suicide after discharge included absurdly high estimates of between a 5 and 70% chance of suicide within days of discharge.^15^ Instead of guessing the probability with the help of hindsight bias,^16^ negligence claims after suicides should hinge on the accepted standard for care of people with various psychiatric disorders, regardless of the presence of known risk factors. Services should be concerned about the adverse consequences of failing to provide an adequate standard of care to any patient they assess or treat, not only those considered to be at high risk.

Pokorny's complaint that the overwhelming number of false positives renders suicide risk assessment unfeasible is just as valid in 2016 as it was in 1983. His finding, which has been replicated in all subsequent studies, poses a challenge to military and civilian mental health services that have been developed around a model of identifying and managing risk. In response, we recommend abandoning attempts to design interventions based on risk stratification and instead aim to provide an adequate standard of care to all of our patients.

## References

[1] Pokorny AD Prediction of suicide in psychiatric patients. Report of a prospective study. Arch Gen Psychiatry 1983; 40: 249–57. 6830404 10.1001/archpsyc.1983.01790030019002

[2] Pokorny AD Suicide prediction revisited. Suicide Life Threaten Behav 1993; 23: 1–10. 8475527

[3] Goldstein RB Black DW Nasrallah A Winokur G The prediction of suicide. Sensitivity, specificity, and predictive value of a multivariate model applied to suicide among 1906 patients with affective disorders. Arch Gen Psychiatry 1991; 48: 418–22. 2021294 10.1001/archpsyc.1991.01810290030004

[4] Madsen T Agerbro E Mortensen PB Nordentoft M Predictors of psychiatric inpatient suicide: a national prospective register-based study. J Clin Psychiatry 2012; 73: 144–51. 21903026 10.4088/JCP.10m06473

[5] Large MM Nielssen OB Risk factors for inpatient suicide do not translate into meaningful risk categories – all psychiatric inpatients are high-risk [Author reply]. J Clin Psychiatry 2012; 73: 1034–5. 22901353 10.4088/JCP.12lr07795

[6] Steeg S Kapur N Webb R Applegate E Stewart SL Hawton K The development of a population-level clinical screening tool for self-harm repetition and suicide: the ReACT Self-Harm Rule. Psychol Med 2012; 42: 2383–94. 22394511 10.1017/S0033291712000347

[7] The Assessment and Management of Risk for Suicide Working Group Va/DoD Clinical Practice Guideline for Assessment and Management of Patients at Risk for Suicide. Department of Veterans Affairs, Department of Defense, 2013.

[8] Kessler RC Warner CH Ivany C Petukhara MV Rose S Bromet EJ Predicting suicides after psychiatric hospitalisation in US Army Soldiers. JAMA Psychiatry 2015; 72: 49–57. 25390793 10.1001/jamapsychiatry.2014.1754 PMC4286426

[9] Szmukler G Rose N Risk assessment in mental health care: values and costs. Behav Sci Law 2013; 31: 125–40. 23296543 10.1002/bsl.2046

[10] Mills PD King LA Watts BV Hemphill RR Inpatient suicide on mental health units in Veterans Affairs (VA) hospitals: avoiding environmental hazards. Gen Hosp Psychiatry 2013; 35: 528–36. 23701697 10.1016/j.genhosppsych.2013.03.021

[11] Olfson M Marcus SC Bridge JA Focusing suicide prevention on periods of high risk. JAMA 2014; 311: 1107–8. 24515285 10.1001/jama.2014.501

[12] Knox KL Stanley B Currier GW Brenner L Ghahramanlou-Holloway M Brown G An emergency department-based brief intervention for veterans at risk for suicide (SAFE VET). Am J Pub Health 2012; 102: S33–7. 22390597 10.2105/AJPH.2011.300501 PMC3496464

[13] Molodynski A Rugkåsa J Burns T Coercion and compulsion in community mental health care. Br Med Bull 2010; 95: 105–9. 20501486 10.1093/bmb/ldq015

[14] Callaghan S Ryan C Kerridge I Risk of suicide is insufficient warrant for coercive treatment for mental illness. Int J Law Psychiatry 2013; 36: 374–85. 23816377 10.1016/j.ijlp.2013.06.021

[15] Rabone *v* Pennine Care NHS Foundation Trust [2012] UKSC 2.

[16] Large M Ryan CJ Callaghan S Hindsight bias and the overestimation of suicide risk in expert testimony [letter]. Psychiatrist 2012; 36: 236–7.

